# The Toxic Impact of Honey Adulteration: A Review

**DOI:** 10.3390/foods9111538

**Published:** 2020-10-26

**Authors:** Rafieh Fakhlaei, Jinap Selamat, Alfi Khatib, Ahmad Faizal Abdull Razis, Rashidah Sukor, Syahida Ahmad, Arman Amani Babadi

**Affiliations:** 1 Food Safety and Food Integrity (FOSFI), Institute of Tropical Agriculture and Food Security, Universiti Putra Malaysia, Serdang 43400, Selangor, Malaysia; rafieh.fakhlaei@gmail.com; 2 Department of Food Science, Faculty of Food Science and Technology, Universiti Putra Malaysia, Serdang 43400, Selangor, Malaysia; madfaizal@upm.edu.my (A.F.A.R.); rashidah@upm.edu.my (R.S.); 3 Pharmacognosy Research Group, Department of Pharmaceutical Chemistry, Kulliyyah of Pharmacy, International Islamic University Malaysia, Kuantan 25200, Pahang Darul Makmur, Malaysia; alfikhatib@iium.edu.my; 4 Faculty of Pharmacy, Airlangga University, Surabaya 60155, Indonesia; 5 Natural Medicines and Products Research Laboratory, Universiti Putra Malaysia, Serdang 43400, Selangor, Malaysia; 6 Department of Biochemistry, Faculty of Biotechnology & Biomolecular Sciences, Universiti Putra Malaysia, Serdang 43400, Selangor, Malaysia; syahida@upm.edu.my; 7 School of Energy and Power Engineering, Jiangsu University, Zhenjiang 212013, China; ar.amani65@gmail.com

**Keywords:** honey, adulteration, sugar adulterants, toxicity

## Abstract

Honey is characterized as a natural and raw foodstuff that can be consumed not only as a sweetener but also as medicine due to its therapeutic impact on human health. It is prone to adulterants caused by humans that manipulate the quality of honey. Although honey consumption has remarkably increased in the last few years all around the world, the safety of honey is not assessed and monitored regularly. Since the number of consumers of honey adulteration have increased in recent years, their trust and interest in this valuable product has decreased. Honey adulterants are any substances that are added to the pure honey. In this regard, this paper provides a comprehensive and critical review of the different types of adulteration, common sugar adulterants and detection methods, and draws a clear perspective toward the impact of honey adulteration on human health. Adulteration increases the consumer’s blood sugar, which can cause diabetes, abdominal weight gain, and obesity, raise the level of blood lipids and can cause high blood pressure. The most common organ affected by honey adulterants is the liver followed by the kidney, heart, and brain, as shown in several in vivo research designs.

## 1. Introduction

The standards of *Codex Alimentarius* [1] defines honey as the natural sweet substance from the nectar of plants or secretions of living parts of the plants that are stored and dehydrated by honey bees to improve its nutritional properties and become consumable for humans.

Honey, traditionally, is used for its anti-aging properties, enhancing the immune system, killing bacteria, treatment of bronchial phlegm, and relieving a sore throat, cough, and cold [2]. Moreover, according to literature, honey represents various pharmacological properties such as anti-inflammatory [3], antioxidant [4], anti-cancer activities against breast and cervical cancer [5], prostate cancer [6], and osteosarcoma [7]. The therapeutic effect of honey on human health can be either oral administration or topical application. In this regard, reference [8] revealed the therapeutic properties of oral administration of honey for the treatment of laryngitis, osteoporosis, gastrointestinal ulcers, anorexia, insomnia and constipation, and liver, cardiovascular and gastrointestinal problems. On the other hand, advantages of topical application of honey are prescribed for eczema, lip sores, sterile and infected wounds, genital lesions, burns, surgery scars, and athlete’s foot [9].

The food industry is one of the critical and fast developing industries worldwide, owing to the tremendous growth of the human population and increased interest of consumers toward the consumption of high-quality products. Moreover, it has been proven that low-quality food products and junk foods may have an adverse impact on consumers’ health [10]. Food adulteration will multiple this risk since the nature of food has been altered. “Food adulteration” is described as the act of intentionally decreasing the quality of food either by adding or swapping low-quality materials or eliminating various important integrant. When the cheaper and low-grade elements are added to an original product threaten the consumer’s health, it is considered and declared “adulterated.” Honey, as one of the most common foods worldwide, also has been subjected to adulteration [11]. Hence, its quality control and safety protocols have become the center of attention of many international committees.

Although honey is recognized as high-quality food, it is more vulnerable to adulteration, mislabeling, and unethical mixing with cheaper and low-grade honey, sugars, and other substances. Moreover, due to its limited availability, proved therapeutic and healing properties, and the increased population concerns regarding their health, there is a rising demand for the natural food product. This increased economic value would make honey a vulnerable adulteration target [12]. Moreover, while honey is a well-known high nutritional value food, it can also be toxic naturally by transferring plant toxins such as pyrrolizidine alkaloids, or because of adulterants that are added into the pure honey by mankind to gain economic profits [13]. Food adulteration has been a major concern for consumers, as it does not only decrease the quality of food products but also results in several adverse health effects. Authentic testing of food and the toxicology of adulterants is required for a value assessment to assure consumer protection against fraudulent activities. According to the regulation set by *Alimentarius* [1], consumers have the right to receive truthful information about the food that they are going to consume. It has also mentioned that honey should not have any added ingredients, any foreign matter, flavor, aroma, or taint absorbed from foreign substances during processing and storage, nor any removal of a particular constituent. Moreover, honey should not be heated or processed to such an extent that its essential composition is changed and its quality impaired. Although honey adulteration is a serious issue worldwide that requires several actions to be solved, there is currently a lack of an effective method to regulate the adulterated honey production [14]. In addition, honey adulteration is a key factor in the honey price fluctuation on the market. Several actions, locally and internationally, have been taken to detect fraud and solve the problem, but there is no actual solution to control the production of adulterated honey [15].

Reference [15] mentioned that sugars could be used in two different ways as adulterants; direct adulteration and indirect adulteration. During direct adulteration, a certain ratio of syrups is added to harvested honey to increase its sweet taste, while in indirect adulteration the bees were overfed with sugar syrups to increase the honey yield in hives. According to Se et al. [13], the most frequent sugar syrups for honey adulteration are high fructose corn syrup (HFCS), corn sugar syrup (COSS), inverted sugar syrup (ISS), and cane sugar syrup (CASS); there is a high preference towards HFCS (from simple isomerization of COSS) according to Se et al. [16]. 

Reference [17] mentioned that in Ethiopia, other adulterants are used for honey, such as water, banana, wheat, and maize syrup or flour. Furthermore, Damto [18] reported the water content, honey processing, and botanical origin as a direct honey adulteration, while admitting that pre-mature harvesting and veterinary drugs, especially an extra dosage, should be considered indirect methods.

According to Jaafar et al. [19], olive oil, milk, honey, saffron, orange juice, coffee, and apple juice are the seven most likely food ingredients to be targeted for economically-motivated adulteration of food (food fraud), as per their article published in the Journal of Food Science. Nowadays, honey adulteration has a major impact on economic loss, not only due to the declining honey quality but also because of the difficulty in the marketing of pure honey. According to Johnson et al. [12], the majority of honey sold on the market is caramelized sucrose that does not have any nutritional value. Since this adulterated material does not have the nutritional value that honey has, consumers have lost sight of the nutritional value of honey.

Some authors have also reported that honey adulteration may cause a reduction in the protein content of honey. In addition, honey adulteration, overheating, or prolonged storage of honey can reduce or eliminate its protein content, as seen in the paper by Lawal et al. [20]. As regards to nitrogen content, reference [21] claimed that there are some variations concerning the nitrogen content of pure and adulterated honey and also pure HFCS, as it ranges from 0.6 for pure honey, 0.3% for 1:1 adulterated honey/HFCS to 0.1% for pure HFCS. The authors mentioned that the nitrogen, proline, potassium, and sodium content of pure honey is much higher than adulterated honey. Abdel-Aal et al. [22] confirmed that the protein content is a reliable factor to investigate the honey adulteration in the samples with less than 30% added sugar. The amino acid content of honey is 50–300 mg/kg, while proline is the most abundant amino acid (50–85%). Particularly, the proline concentration ratio (180 mg/kg) is an indication value to differentiate between natural and adulterated honey [23], which is more accurate in comparison to the protein content [24,25].

Unfortunately, there would be a risk of detecting pure honey as the adulterated type if C4 plants are being used in honey production with other plants since it causes the product to exceed the δ^13^C value [23]. Plants are classified into three groups, in accordance with the photosynthesis pathway: C3 plants and C4 plants can bio-fixate carbon dioxide into a 3-carbon compound by using the Calvin cycle and a 4-carbon compound via the Hatch-Slack cycle, respectively, while crassulacean acid metabolism (CAM) plants can use both cycles [26].

However, the δ^13^C value might reduce by the addition of a sugar adulterant (below 23.5%) and mislead the detection of honey adulteration [27]. Therefore, both C isotope ratios of raw honey and protein fractions (δ^13^C_honey_ and δ^13^C_protein_) should be considered to detect the honey adulteration by the addition of sugar [28].

Adulterants are any substances that are added to the original and pure product. Honey can be adulterated directly (addition of adulterants) [28], indirectly (bee-feeding) [26,29], or by blending it with other cheap honey [30,31], which will be discussed further below. The quality of honey is closely related to its impurities and adulterants.

The adverse health impact of honey adulteration on consumers may lead to increased blood sugar, followed by the release of the insulin hormone and type II diabetes, abdominal weight gain and obesity, a rise in the blood lipid levels, and high blood pressure [32]. Furthermore, adulterants can affect internal organs, potentially causing a fatty liver [13], acute and chronic kidney injury [33] and elevate visceral fat pads and total body fat, which can lead to death [12,15].

Honey adulteration is due to various reasons, such as adding sugars to enhance the taste based on the consumer’s preference or gaining financial profits by mixing cheap and low-quality honey to the expensive honey to increase the yield. As we have checked various research databases such as Scopus, the Web of Science, and Google Scholar, there is not any significant study on the toxicological effect of honey adulteration on human health up to this date. Hence, this review paper can be a foundation for scientists and researchers to investigate commercial honey adulterants and the disadvantages of adulterated honey on consumers’ health. This review comprehensively covers the outline regarding different types of adulterants, methods of honey adulteration, and the toxic impact of honey adulteration on internal organs via various in vivo and in vitro studies.

### 1.1. Honey

Honey is defined as the excretions of insects sucking on the living parts of plants. Honeybees are the most well-known plant-sucking insects and can collect and transform honey, and deposit, dehydrate, store and leave honey in the honeycomb to ripen and mature. Honeybees collect pollen and nectar from a variety of flowering plants and convert it into the wax and honey [34]. Only worker honeybees forage for food, consuming as much nectar from each flower as they can. After foraging, worker honeybees return to the hive/comb and pass the collected nectar to the other worker honeybees. This worker holds the nectar on her tongue until the liquid evaporates, creating honey. The honey is then stored in a cell within the hive/comb.

### 1.2. Type of Honey

Reference [5] and Alvarez-Suarez et al. [35] classified honey according to its origin as follows:

(1) Blossom honey: the main source of this honey is the nectar of flowers such as linden, clover, citrus, cotton, thyme, and acacia honey.
(2) Honeydew honey: the source of this honey is the “honeydew” (*Rhynchota* genus insects pierce plant cells, ingest plant sap, and then secrete it again) collected by bees. A typical example of honeydew honey is pine, oak, fir, and leaf honey.
(3) Monofloral honey: named according to the plant that the bees that have produced the honey forage predominantly.
(4) Multifloral honey (polyfloral): the source of this honey is several botanical flowers, with none of them predominant. Meadow blossom honey and forest honey are classified in this category.

All these classifications indicate the quality and physicochemical properties of honey. The honey composition and quality varies according to the botanical origin, geographic area, and harvesting season [26]. Moreover, honey can be classified based on the bee species (stingless and honeybee) as mentioned in the next sections.

#### 1.2.1. Stingless Bee Honey

**Figure 1:**
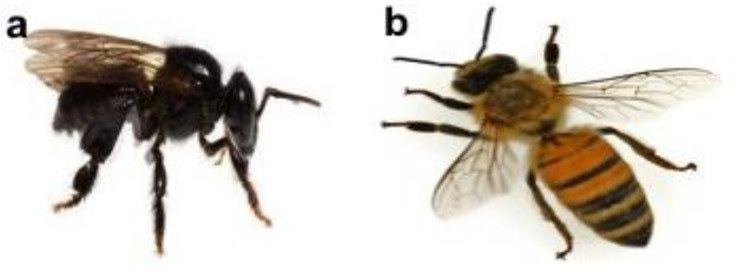
Different types of bees: (**a**) *Heterotrigona itama* bee, (**b**) *Apis mellifera* bee.

Among all five genera of the stingless bee, there are the only two types of stingless bees that produce honey, namely, *Melipona* and *Trigona* [36]. As a result of the previous study, stingless bee honey, in comparison to sting bee honey, has been shown to be higher in moisture content, acidity, and lower in diastase activity [37]. Furthermore [5], stingless bees can be differentiated from sting bee in terms of morphology (absence of sting) (Figure 1a), collection of nectar, their short harvest distance, and their honeycomb-less hives.

These species of bees are not dangerous to humans and are very active as compared to other bees. Among all these species, only two types, named *Heterotrigona itama* (*H. itama*) and *Geniotrigona thoracica*, are kept by beekeepers, as they produce a higher volume of honey as compared to the others. In this regard, Se et al. [16] stated that *H. itama* honey has significantly higher physicochemical values when it comes to moisture content, water activity, free acidity and color intensity, and better antioxidant properties in comparison with other types of honey. Furthermore, Bakar et al. [38] mentioned that *H. itama* honey is less concentrated, with a more sour taste and aroma in comparison with other types of honey. Moreover, stingless bee store honey in a honey pot as opposed to a honeycomb.

#### 1.2.2. Honeybee (*Apis*)

Among all recognized *Apis* species, there are only two commercially used by mankind, namely *A. mellifera* and *A. cerana*. This is due to the behavioral limitation of other species, such as dwarf and giant honeybees, which practice open-air nesting and cannot be kept in manmade hives. A comparison between these two species, *A. mellifera* generally is more productive in producing honey compared to *A. cerana* [3]. Furthermore, honeybees (Figure 1b) store honey in a honeycomb.

Honey from honeybees is a viscous, high-nutrient food. Its most important contents are fructose and glucose (80% ± 2), water (16% ± 1), ash (0.2%), and amino acids (<0.1%), while enzymes, vitamins, phenolic compounds, and other substances are present in trace amounts [39].

### 1.3. Worldwide Honey Production and Consumption

Globally, the honey market was worth more than USD 7.5 billion in 2018, and it will reach USD 10.5 billion by 2025 [5]. As regards the geographical production distribution, the Asia Pacific had the biggest market share in 2018. China is the top producer, with a production volume of 490.84 K (27.5% of the global production), followed by Turkey with a production volume of 105.53 K (5.9% of the global production) and Iran with a production volume of 80.56 K (4.5% of the global production) [40]. This noticeable geographical production growth can be attributed to the regional rise in the supply and demand chain, and also to the growing awareness of consumers regarding the benefits of honey and its advantages over normal sugar. Furthermore, the United States placed the fourth among the top producers of honey, with a production volume of 73.43 K (4.1% global production) in 2016. The production of honey in the European Union (EU) has been increased moderately (250,000 tons/year in 2015), which established the EU as the next largest honey supplier after China [40]. The main producers in the EU are Romania, Spain and Germany followed by Hungary, France, Greece, and Poland [41].

### 1.4. Regulation Related to Honey

The authors in [40] established two criteria regarding the sugar content of honey: (1) the total concentration of fructose and glucose should not be less than 60% (*w*/*w*) for blossom honey, and not less than 45% (*w*/*w*) for honeydew honey and related blends; (2) the sucrose concentration should not exceed 5% (*w*/*w*) (Table 1). Concerning the above information, CODEX and INTERGOVERNMENTAL [14] reported that 14% of honey from EU and non-EU was considered adulterated by sugar.

In this regard, the Australian New Zealand food standard [41] proposed the regulation that a food that is prescribed as ‘honey’ must contain no less than 60% reducing sugars and no more than 21% moisture.

Based on the established China honey standard, Reference [42] mentioned there are additional requirements for cane sugar content in Eucalyptus, citrus, clover, Lychee, and wild osmanthus honey, namely, not less than 10 g/100 g, while in other honey it cannot be less than 5 g/100 g. Moreover, the zinc content in honey is also limited to ≤25 mg/kg.

### 1.5. Nutritional Value of Honey

Based on the nutritional value of honey, this healthy natural food contains not only fructose, glucose, and water, but also trace amounts of valuable ingredients, such as flavorings, vitamins (B_1_, B_2_, B_3_, B_5_, B_6_, B_9_, C, and K), minerals (Na, Ca, K, Mg, P, Se, Cu, Fe, Mn, Cr, Zn), enzymes and antioxidant [14] (Table 2 and Table 3).

Honey is one of humankind’s oldest food products that preserve human health and shields them from various diseases, such as cancer, a cold, sore throat, etc. [34,44]. There is a study that shows that fructose in pure honey tends to lower blood glucose in animal models of diabetes [18]. The mechanism of reducing blood glucose is as follows: it reduces the rate of intestinal absorption [45,46], extends the gastric emptying time [47], and reduces food intake [48]. Fructose stimulates glucokinase in hepatocytes, which play an important role in the uptake and storage of glucose as glycogen by the liver. Hence, glucose, which is present in combination with fructose in honey, enhances the absorption of fructose and promotes its hepatic actions through its enhanced delivery to the liver [49].

Consumption of honey as a food or a natural sweetener can boost the human body’s energy level. Honey has high antioxidant activity and antimicrobial properties and can be used for healing wounds, treat obesity, diabetes, and cancers [34].

Several studies have revealed that honey has biological properties and health benefits on the human body, as discussed here. An in-depth study by [7] stated that natural honey has a significant effect on reducing cholesterol (7%), triglycerides (2%), C-reactive protein (7%), and homocysteine (6%).

The therapeutic effect of honey on human health can be either through oral administration or topical application. In this regard, Al-Waili et al. [50] revealed the therapeutic properties of oral administration of honey for the treatment of laryngitis, osteoporosis, gastrointestinal ulcers, anorexia, insomnia and constipation, and liver, cardiovascular and gastrointestinal problems. On the other hand, advantages of the topical application of honey are prescribed for eczema, lip sores, sterile and infected wounds, genital lesions, burns, surgery scars, and athlete’s foot [9].

The therapeutic effect of honey can be tested on animals and then extrapolated to humans. Therefore, according to an in vivo study done by [10] on rats early mortality and histopathology result, several health modifications have been observed after the consumption of commercially adulterated honey: weight gain, an abnormal parameter in renal and hepatic function, a rise in the level of circulating triglycerides, cholesterol and glucose levels, fat deposition augmentation and severe organ toxicities. Moreover, this research is in agreement with the study done by Samat et al. [15] on rats, which showed that adulterated honey consumption may be harmful and cause liver and kidney dysfunction.

## 2. Honey Adulterants

Low-cost sugars and commercial syrups are common substances for honey adulteration. Ismail and Ismail [51] described well-known adulterants from sugar cane and sugar beet such as corn syrup (CS), HFCS, glucose syrup (GS), sucrose syrup (SS), inverted syrup (IS), and high fructose inulin syrup (HFIS). Adulteration of honey by sugars alters the chemical and biochemical properties of honey, such as the enzymatic activity, electrical conductivity, and specific compounds contents [13].

**Figure 2:**
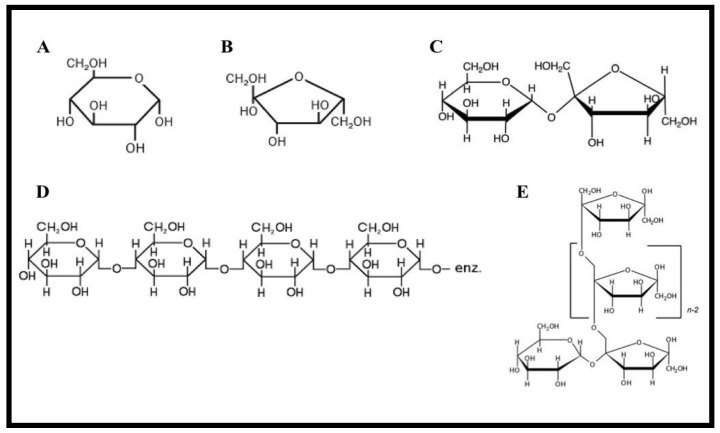
Chemical structures of widely used sugar adulterants in honey. (**A**) Glucose, (**B**) Fructose, (**C**) Sucrose, (**D**) Rice syrup, and (**E**) Inulin syrup.

Honey adulterates are selected based on the following three factors: the specific region of origin, the economic benefits and the sugars or sweeteners accessibility. A well-known example is employing rice and wheat syrup extraction as adulterants in Turkey and France [13]. The plant syrups can be harvested from heating vegetable juices or partial enzymatic hydrolysis [31,43,52,53]. According to [54,55], European countries adulterate the honey with HFIS. In this section, common sweeteners that are used in commercial honey adulteration will be introduced and their health impact discussed. The chemical structures of these widely-used sugar adulterants are presented in Figure 2.

### 2.1. Cane Sugar

Cane sugar is sucrose consisting of two sugar molecules monosaccharides (glucose and fructose). Glucose and fructose are monosaccharides with an identical chemical formula (C_6_H_12_O_6_) but a different chemical conformation, linked together by a weak glycosidic bond to produce sucrose (C_12_H_22_O_11_), a disaccharide. Generally, cane sugar is obtained by extracting juice from the sugar cane, a perennial C4 grass, followed by purification by chemical and physical means, evaporation to remove the water and separation of the sugar crystal [56]. Cane sugar originates from plants with a C4 metabolic pathway (Hatch-Slack cycle), while nectar originates from the C3 metabolic pathway (Calvin cycle).

In another study [57], cane sugar was used in both direct and indirect adulteration of honey. During direct adulteration, 10, 20, and 40% of syrup were added to the honey sample; for the indirect method, the bees were fed with syrup.

Concerning the toxicity of sugar, the lethal dose (LD_50_) is a useful tool to measure the short-term toxicity and causes the death of 50% of a test animal population. Hence, the acute oral LD_50_ of cane sugar in rats is 29,700 mg/kg BW, almost 30 g/kg, which placed this sugar into a particularly safe scale [30].

### 2.2. Corn Syrup

Corn syrup, or high fructose corn syrup (HFCS), is a viscous, odorless, and colorless liquid that is much denser than water. Corn syrup is a liquid sweetener derived by cornstarch hydrolysis, which is used as a sweetener in foods [58]. Based on its fructose content, corn syrup is classified as: HFCS-42 (42% fructose), HFCS-55 (55% fructose), HFCS-90 (90% fructose) [59]. The fructose from high fructose corn syrup cannot be used directly to generate energy and has to be stored in the liver as fat or glycogen. Hence, the extreme amount of fructose from HFCS cannot be processed beneficially in the body [60]. The LD_50_ of rare sugar syrup, which is obtained from HFCSm is 15,000 mg/kg BW for rats with no abnormalities; in humans, the acute non-effect level, which caused diarrhea, was estimated as 0.9 g/Kg BW as a dry solid base [61].

### 2.3. Palm Sugar

Palm sugar is extracted from the flower buds of the palm. It is a natural sweetener undergoing minimum steps during the chemical-free procedure. One study [62] reported that the major carbohydrates in palm sugar were sucrose, followed by glucose and fructose. The significant advantage of palm sugar is the lack of a blood sugar spiking effect, owing to its low glycemic index (~35). The most popular honey adulterant in India is jaggery syrup, which is prepared by the evaporation of palm tree extraction the evaporation of the sap of palm trees [63]. While sucrose and glucose are the main sugar components of palm sugar, the LD_50_ for sucrose is 29,700 mg/kg BW and for glucose 25,800 mg/kg BW for a rat [64].

### 2.4. Invert Sugar

Invert sugar (IS) is produced by cleavage of the sucrose into its monosaccharides building blocks, fructose, and dextrose. The inversion procedure is usually performed by heating the sucrose syrup in the presence of acids, alkali, or invertase [58]. The sugar content of IS originates from beet and cane plants, mimicking the pure honey sugar profile [65]. Invert sugar has been widely used in beverages and food industries for making non-crystallized cream, jams, artificial honey, and liquid sugar [16].

Inverted beet syrup is one of the most well-known adulterants, which can be tailored to mimic the natural sucrose (glucose-fructose) profile of honey and, as beet is a C3 plant, it is usually difficult to detect. In one study, various quantities of inverted beet syrup were added to the pure honey samples of clover, orange, and buckwheat [66]. Invert sugar is a generally accepted safe substance and it does not present toxic effects. It is recommended to take precautions in patients that present diabetes mellitus and also in patients with the rare hereditary problems of fructose intolerance, glucose-galactose malabsorption, or sucrase-isomaltase insufficiency [67].

As inverted sugar is a mixture of fructose and glucose, the LD_50_ value for this sugar is adopted from its structural sugars; the LD_50_ for fructose and glucose are 25,800 mg/kg BW and 29,700 mg/kg BW, respectively [68].

### 2.5. Rice Syrup

Rice syrup (RS), a product of rice polysaccharide hydrolysis, originating from a C3 plant (similar to beet syrup), is one of the most popular honey adulterants in China [58].

Rice syrup contains three sugars: maltotriose (52%), maltose (45%), and glucose (3%). Since maltose is two molecules of glucose and maltotriose is three molecules of glucose, rice syrup acts like 100% glucose inside the body. Honey adulterated with RS has recently emerged on the honey market. Rice syrup is a C3 syrup adulterant that follows a similar Calvin cycle of photosynthesis as natural honey [28]. Thus, rice syrup as a honey adulterant is a critical issue that affects quality assurance and food safety [69].

The unethical substitute brown rice syrup with HFCS in some organic foods has raised researchers’ concerns due to its high arsenic content [70]. Baby formulas that contain organic brown rice syrup (OBRS) have an increased arsenic level, above the drinking water standard as per the research led by [71], and there is no regulation to govern this particular scenario. Rice syrup acts as glucose inside the body; the LD_50_ of rice syrup is the same as glucose, which is 25,800 mg/kg BW [72].

### 2.6. Inulin Syrup

Inulin is naturally occurring polysaccharide, belonging to a class of fructans. These nutritional fibers are a chain of fructose residues linked to glucose at the end of the chain. The linkage arrangements of the fructose molecules determine the fructan type. For example, in the case of inulin, the chain of β2-1 linked fructose has been terminated by glucose. The common source of this polysaccharide are wheat, onion, bananas, garlic, asparagus, sunchoke, and chicory [58]. Furthermore, [70] added different proportions (5, 10, and 20%, *w*/*w*) of high fructose inulin syrup to a nectar honey sample to intentionally simulate honey adulteration.

Classical toxicology tests are difficult to apply to inulin, which is a micro ingredient. Although some high dose animal tests have been performed, none have revealed any toxic effects [56]. Thus, the LD_50_ values for fructose, glucose and sucrose are 25,800 mg/kg BW, 29,700 mg/kg BW and 29,700 mg/kg BW, respectively [73].

## 3. Adulteration Method

Commercial honey adulteration is typically classified as direct, indirect, and blending [58], as shown in Figure 2. As discussed earlier, the authors in one paper [60] categorized the honey adulteration into two types; direct and indirect. The direct addition of sugar syrups is a post-production procedure of adding certain ratios to increase honey sweetness [13]. Meanwhile, indirect adulteration occurs by overfeeding the bees during the main nectar period with honey, chemicals, and industrial sugars to recover more honey from hives [28]. Blending is another honey adulteration procedure, which can be explained as mixing pure and high-quality honey with cheap and low-quality honey [19,31]. However, the syrup or sugar residues of some reported studies are identical to the natural residues in the honey. Therefore, the detection of these adulterants have proved difficult, and scientists have to discover new methods to distinguish the differences between pure and adulterated honey [32].

### 3.1. Direct Adulteration

Direct adulteration of honey is commonly performed by the direct addition of a certain amount of sucrose syrup into the honey. The source of sucrose syrup could be sugar beet, HFCS, maltose syrup, or industrial sugar syrups (glucose and fructose) obtained from heat, enzyme, or acid treatment of starch [74]. Direct adulteration causes harm to consumers and pure honey producers [28].

According to a study done by [31], to make direct adulterated honey, pure honey was mixed with different concentrations (7%, 15%, and 30%) of date and inverted sugar syrups. The authenticity of the samples was estimated by applying a multivariate analysis (PCA and LDA) into the physicochemical and rheological analysis. Amiry et al. [75], on the other hand, performed direct adulteration of honey with synthesized sugar. Adulteration was done by mixing glucose powder with distilled water and then adding it to the pure honey in laboratory design experiments. The honey adulteration was detected by an optical microfiber sensor in this research.

Moreover, Irawati et al. [76] added the two rice syrups directly with a ratio of 1.2:1 to the pure honey to provide a mixture with a viscosity close to the honey. Furthermore, three-dimensional fluorescence spectra (3DFS) and multivariate calibrations were employed as a detection method for honey authenticity.

### 3.2. Indirect Adulteration

Indirect adulteration of honey is the incorporation of sugars into honey via bee-feeding [77]. In this manner, low-quality honey, chemicals, and industrial sugars were incorporated into the honey during a natural process that happened in the bee’s digestive system [78]. During indirect honey adulteration, an extreme amount of sugar syrup was fed to the bee colonies in the main nectar flow period [31].

One paper [79] described the indirect adulteration as follows: the colonies were settled in empty beehives with bees, brood, and honey frames. Standard bee-feeding methods were applied in the early spring [77]. The sucrose syrup (1:1.5 *w*/*w*, water: sugar) as adulterants were presented to each colony to ensure the growth and strength of the forage worker bee population of colonies for the main nectar flow season. The honey and honeycomb frames of all colonies were taken at the end of the nectar flow season [80]. After settling the bees in the hives, no more syrup was provided to the colonies.

Furthermore, other researchers [81] developed a method analyzing the biochemical properties to distinguish indirect sucrose syrup (SS) adulterated honey from pure blossom honey. According to the result of this study, adulteration of honey by SS has no significant effect on the content of honey, since over 95% of the fed SS to the bees was converted to fructose and glucose. During the initial step of the bees’ SS feeding, the sucrose content was hydrolyzed into its building blocks. However, after extending the intensive feeding duration, the bees start to convert and store the glucose into maltose and maltotriose oligosaccharides [82].

The amount of sucrose present in pure honey owes little to the activity of invertase enzymes present in pure honey. This is the enzyme in charge of breaking down the sucrose into its building blocks and reducing the sucrose content of pure honey. Hence, it can be concluded that high sucrose levels in a honey sample might be due to the adulteration of honey [83].

According to the experiment done by [18] on indirect adulteration of honey, prolonged SS feeding will have identical results as with artificial direct adulteration. Furthermore, the extended prolonged SS bee-feeding reduces the nutritional properties and quality of the honey in the same manner of direct honey adulteration [30].

### 3.3. Blending

In this method, high quality (pure and rare) honey is mixed with cheaper honey that has lower quality and nutrition. Adulteration of pure honey with synthetic honey has become much more prevalent in recent years [30].

In China and Venezuela, a well-known fraud in the honey industry consists of blending the costly acacia honey with rape honey (cheaper honey) to increase the marketing profit since the light amber color of rape honey is highly similar to that of the yellow-colored acacia honey [76]. Acacia honey originates from *Robinia pseudacacia* blossoms and is commonly consumed nectar honey because of its mild aromatic and clear aspect and light yellowish color, and because it does not crystallize [32]. In contrast, rape honey is sweeter, with a light amber color, and easily crystallizes [84]. Hence, as the collection of *M. favosa* honey has steadily increased since 1985, it is the perfect target for blending with the cheaper honey of *A. mellifera* [32]. In this study, acacia honey was blended with different concentrations (5–50%, *w*/*w*) of rape honey, and the simple method of liquid chromatography-electrochemical detection (LC-ECD) was used to detect the adulteration.

Since the honey adulterants and adulteration methods have been discussed earlier, detection methods to investigate the authenticity of honey become the priority in the food safety of honey. These detections methods must be capable of distinguishing between pure and adulterated honey to assist the authorities in regulating the market and protect the consumer’s right.

## 4. Detection Method in Honey Adulteration

The addition of sugar to honey is the common fraud in honey adulteration. Sugar addition always has been coupled with thermal treatment to produce a homogenous mixture to sell it as pure honey to the consumers. During applying heat to the honey, HMF can be produced as an intermediate compound via the Maillard reaction. So, the amount of HMF is an indicator of honey adulteration. There is a possibility of HMF formation at low temperature and can be elevated by the increase of applied temperature or storage [85]. High-performance liquid chromatography (HPLC), gas chromatography with mass spectrometry (GC-MS), micellar electrokinetic capillary chromatography (MEKC), and voltammetry are among the methods that applied for the determination of HMF in adulterated honey [86]. A physiochemical analysis is another application to differentiate pure honey from adulterated honey. Honey adulterated with fructose and saccharide showed a lighter color (*L* values), while pure honey shows darker red (*a* values) and yellow (*b* values) color. Moreover, there is a decreasing trend in pH values and an increasing trend in water activity (a_w_) with the addition of fructose and saccharides to the pure honey [87].

In addition to HMF, diastase enzymatic activity can also be used for detecting sugar adulteration [88]. Enzymatic activity (diastase and invertase) is one of the simplest and most applicable analytical methods, though it is not always conclusive and must be paired with other analytical methods to generate a reliable result. As a comparison between these two enzymatic activities, invertase is a better marker for the quality control of honey, since it degraded much faster during the heating up procedure of honey, compared to amylase [14]. Moreover, Differential Scanning Calorimeter (DSC) can be used to determine the thermal properties of honey influenced by the direct addition of sugar syrup adulterants [89]. Furthermore, the conventional polymerase chain reaction (PCR) can detect several genes of rice molasses, which has been used in the direct adulteration of honey. Furthermore, the standard curve attained from real-time PCR can quantify the rice molasses’ DNA amount and calculate the exact level of adulterants [90].

Several detection techniques have been developed in recent studies with the aim of detection of direct honey adulterated with sugar, such as high-performance anion-exchange chromatography with pulsed amperometric detection (HPAEC-PAD) for the detection of CS and HFCS, HPLC, and stable carbon isotope ratio analysis (SCIRA) for the detection of HFS [91], GC-MS for the detection of HFCS [32], cavity ring-down spectroscopy (CRDS) and isotope ratio mass spectrometry (IRMS) for the detection of CS [92], three-dimensional fluorescence spectroscopy (3DFS) for the detection of RS [93], Raman spectroscopy for the detection of HFCS [77], matrix-assisted laser desorption/ionization mass spectrometry (MALDI-MS) for the detection of IS and CS [94] and headspace-gas chromatography coupled to ion mobility spectrometry (HS-GC-IMS) for the detection of CS [95].

However, ultrahigh-performance liquid chromatography coupled with quadrupole time-of-flight mass spectrometry (UHPLC/Q-TOF-MS) was applied for indirect adulteration of honey, through bee feeding with multi-class sugar syrups, such as HFCS, RS, and IS [96]. Hence, the liquid chromatography-electrochemical detection (LC-ECD) [69] and laser-induced breakdown spectroscopy (LIBS) [97] methods were used for the detection of acacia honey blended with rape honey.

The chemometric analysis is widely applied to the detection method as a helpful tool for the reduction of samples’ complexity and classifies them into groups based on their similarities. The most commonly used chemometrics techniques are the principal component analysis (PCA) [98], orthogonal projection to latent structure discriminant analysis (OPLS-DA) [97], partial least square-linear discrimination analysis (PLS-LDA) [96], and partial least squares regression (PLSR) [94]. Table 4 represents the most recent detection methods for the identification of honey adulteration based on sugar adulterants.

## 5. Adverse Health Impact of Honey Adulteration

The adverse health impacts of consuming adulterated honey on human health are not completely established yet due to an absence of systematic and scientific studies and lack of public awareness. Pure honey showed significantly lower toxicity due to containing simple sugar (glucose and fructose) and other essential nutrients such as proteins, antioxidants, and minerals [98]. While honey has an antibacterial effect, helping to fight common cold and some digestive problems, the mixture of inverted sugar or jaggery can sometimes restrict the antibacterial properties of honey and lead to stomach disorders [78]. Adulteration harms consumers’ health, which may cause increased blood sugar followed by the release of the insulin hormone and type II diabetes, abdominal weight gain, and obesity, a raise in the level of blood lipid, and high blood pressure [99].

Consumption of glucose from sugar-adulterated honey may elevate insulin secretion. Insulin activates the plasma membrane enzyme system with the properties of NADPH-oxidase resulting in not only the production of H_2_O_2_ and fructose but also increases uric acid in humans and rodents [13]. Uric acid generation in the body has unfavorable effects, namely, the inability to scavenge lipophilic radicals and breaking the radical chain propagation within the lipid membrane [100]. Under the same circumstances, glucose and fructose of sugar produce ROS through various mechanisms in the body, which is detrimental toward human health and causes chronic diseases such as atherosclerosis, diabetes, obesity, hypertension, coronary artery diseases, and finally heart failure [101]. Glucose that is either naturally produced in the body or added to foodstuff can be detrimental to humans.

Furthermore, the authors of one paper [102] investigated the short-term (two weeks) and long-term (16 weeks) effects of two different brands of honey consumption in the Malaysian market using male Sprague Dawley rats. The finding was illustrated that a total of five rats from both adulterated honey groups showed early mortality and many abnormal signs developed compared to rats fed with natural honey (pineapple honey) as a control. The abnormalities in the adulterated honey group represent significant body weight, fat pads, and BMI, and serum lipid profile (triglycerides, cholesterol, and glucose level) drastically increases. Since glucose and fructose can instantly convert to energy inside the cells, they represent a lower glycemic index compared to sucrose [15]. These results could indicate that long-term consumption of adulterated honey has a harmful impact on human health, requiring local and international authorities’ actions to control and regulate this matter.

The kidney serology and toxicology study of rats that consumed adulterated honey for 16 weeks showed kidney damage due to losing their capability to expel creatinine and urea from the serum [50]. In another in vivo study [15], it was proved that a long-term high sucrose diet may lead to the increment of both urea and creatinine. Meanwhile, Li et al. [103] stated that prolonged consumption of HFCS would lead to rat’s glomerular filtration failure.

According to a recent study by Arise and Malomo [104] on rats, hypercholesterolemia, hypertriglyceridemia, and hyperinsulinemia are caused by prolonged feeding of any sugar syrup such as sucrose or fructose, which leads to animal death. Furthermore, Ajibola et al. [34] reported the synthesis of triacylglycerides in the liver as a result of increasing the gene expressions of lipogenesis enzymes such as acetyl CoA carboxylase and fatty acid synthase.

The morphological alteration in response to diseases and toxic compounds are often critical to our understanding of normal and abnormal function. Histological studies facilitate a new chapter of science into the architecture of tissue, which is biologically and clinically valuable. Larson-Meyer and Willis [105] observed a pyknotic nucleus with a reduced diameter in the liver of the tilapia fish—*Oreochromis niloticus*—exposed to different dilutions of sugar cane vinasse (1%, 2%, 5%, and 10%). These results could explain the effect of the consumption of the high amount of fructose in adulterated honey, which leads to a rise in the fasting serum uric acid level in the liver, followed by increased insulin resistance (caused diabetes), NAFLD score, lobular inflammation and steatosis grades [106]. On the other hand, fructose intake stimulates de novo lipogenesis (DNL) in the liver, which prevents fatty acid oxidation and leads to accumulation of fatty acid in the liver [107].

In other research [107], rats fed with sugar adulterated honey exhibited a pale reddish color, exceptional kidney size, and observed nodules outside the kidneys. There was also an abnormal difference in the appearance of the liver, with slight discoloration to brown, particularly in the middle of this organ’s surface. In detail, some of the livers showed a roughly brown surface and their sizes were smaller, with whitish micronodules on the entire liver surface. Regardless of weight differentiation, the relative weight of the kidneys and lungs from rats fed with adulterated honey showed a significant increase compared to control rats. Moreover, the heart and brain from the rats fed with adulterated honey exhibited significant decreases [15]. These types of intensive investigations may be harmful toward the human body, and authorities should avoid it in the future.

Natural honey possesses some beneficial effects, such as lower total cholesterol and LDL in healthy overweight human subjects, while consumption of 3 to 20% of dietary fructose caused the elevation of total cholesterol and LDL by 9% and 11%, respectively [15]. In this regard, the addition of sugar to honey could be critical toward human health.

Although investigating the direct effect of adulterated honey on human health is an impossible process, it is a crucial procedure and needs ethical approval [108]. To our knowledge, no studies investigated the toxic effect of adulterated honey on humans. However, more animal studies need to be done on the toxic impact of adulterated honey in order to correlate the results to humans.

Table 5 represents the recent studies that investigated the in vivo effect of various adulterants on internal organs. Several sugars were tested on the internal organs in Sparague Dawley rats, such as commercial honey, sucrose, high fructose corn syrup, sugar syrup, and fructose; these caused liver and kidney damage [102], elevated blood urea and creatinine [15], kidney failure [103], elevated visceral fat pads and total body fat, which caused the animal death [104] and acute and chronic kidney injury leading to renal failure [34] and hyperglycemia [12], respectively. Moreover, sugar cane vinasse fed to *Oreochromis niloticus* fish caused liver damage [33]. Furthermore, feeding broiler chicken sugar cane extract resulted in a higher value of the intestinal villus height and area [106].

## 6. Conclusions

The significant impact of honey adulteration on market loss, reducing the quality of honey, shows the importance of studies to investigate different honey adulterants, adulteration methods, and detection methods. Moreover, this fraud has an adverse impact on the honey production industry and market by reducing the trust of consumers on this valuable product. According to this review, all six studied sugar adulterants (cane sugar, corn syrup, palm sugar, invert sugar, rice syrup, and inulin syrup) have health disadvantages toward human health based on their LD_50_ value and internal organ toxicology. The kidney and liver are the main organs that fail due to the consumption of sugar-adulterated honey. Diabetes, CKD, and AKI are the direct results of honey adulteration stated by in vivo histological examination. These diseases have a noticeable impact on human daily life and social health. Hence, the discussed detection methods can be used to identify honey adulteration via chemometric methods. While these techniques require specific laboratory knowledge and equipment, rapid and accurate detection methods, with ease of public accessibility to save customer’s expenses and time, and guarantee their safety, are absent. As has been discussed in this review, more prompt actions must be taken by authorities to prevent the production, trading, and marketing of adulterated honey and discover the other harmful honey adulterants available on the market. The overall result demonstrates that honey adulteration is a threat to food safety, food security, and ecological sustainability of this important and valuable product.

## Figures and Tables

**Table 1:** Essential composition and quality factors of pure honey (adopted from Wei et al. [43]).

Component	Honey	Value
1. Moisture content	Honey that is not listed below	<20%
	Heather honey (*Calluna*)	<23%
2. Sugar content		
a. Fructose + Glucose content	Honey that is not listed below	>60 g/100 g
	Honeydew honey and its blends with blossom honey	>45 g/100 g
b. Sucrose content	Honey that is not listed below	<5 g/100 g
	Alfalfa (*Medicago sativa*), *Citrus* spp., False Acacia (*Robinia pseudoacacia*), French Honeysuckle (*Hedysarum*), Menzies Banksia (*Banksia menziesii*), Red Gum (*Eucalyptus camaldulensis*), Leatherwood (*Eucryphia lucida*), *Eucryphia milligani*	<10 g/100 g
	Lavender (*Lavandula* spp.) and Borage (*Borago officinalis*)	<15 g/100 g
3. Water-insoluble solid content	Honey that is not listed below	<0.1 g/100 g
	Pressed honey	<0.5 g/100 g

**Table 2:** Nutritional composition of pure honey.

Nutrition	Blossom Honey	Honeydew Honey
	Range	Range
Water	15–20	
Total sugars		
*Monosaccharides*		
Fructose	30–45	28–40
Glucose	24–40	19–32
*Disaccharides*		
Sucrose	0.1–4.8	0.1–4.7
Others	2.0–8.0	1.0–6.0
*Trisaccharides*		
Erlose	0.5–6.0	0.1–6.0
Melezitose	NA	0.3–22
Others	0.5–1.0	0.1–6.0
Minerals	0.1–0.5	0.6–2.0
Amino acids, proteins	0.2–0.4	0.4–0.7
Acids	0.2–0.8	0.8–1.5
pH value	3.2–4.5	4.5–6.5

Data in g/100 g of honey, adopted from [48].

**Table 3:** Chemical elements found in pure honey.

Minerals	Amount (mg/100 g)	Vitamins	Amount (mg/100 g)
Sodium (Na)	1.6–17	Thiamine (B1)	0.00–0.01
Calcium (Ca)	3–31	Riboflavin (B2)	0.010.02
Potassium (K)	40–3500	Niacin (B3)	0.10–0.20
Magnesium (Mg)	0.7–13	Pantothenic acid (B5)	0.02–0.11
Phosphorus (P)	2–15	Pyridoxine (B6)	0.01–0.32
Selenium (Se)	0.002–0.01	Folic acid (B9)	0.002–0.01
Copper (Cu)	0.02–0.6	Ascorbic acid (C)	2.2–2.5
Iron (Fe)	0.03–4	Phyllochinon (K)	0.025
Manganese (Mn)	0.02–2		
Chromium (Cr)	0.01–0.3		
Zink (Zn)	0.05–2		

Adopted from Ajibola, Chamunorwa, and Erlwanger [34].

**Table 4:** Summary of analytical approaches of articles related to the honey adulteration with sugar.

Adulterants	Targeted	Non-Targeted	Reference
Rice molasses	Conventional and real-time PCR	-	[98]
Sugar syrup	DSC	PCA	[91]
HFS	HPLC; SCIRA	-	[90]
CS and HFCS	HPAEC-PAD	-	[32]
RS	3DFS	PCA	[17]
CS	HS-GC-IMS	OPLS-DA	[77]
HFIS	GC-MS	-	[96]
HFCS	RAMAN spectroscopy	PLS-LDA	[92]
IS and CS	MALDI/MS	-	[94]
CS	CRDS; IRMS	-	[95]
RS, HFCS, CS, IS	UHPLC/Q-TOF-MS	-	[93]
Rape honey	LC-ECD	PCA	[69]
Rape honey and HFCS	LIBS	PLS	[97]

**Table 5:** Different in vivo studies of sugar adulterant impact on an internal organ.

Type of Sugar	Animal Type	Affected Organ	Reference
Inverted sugar	Human being	Stomach disorder	[109]
Commercial honey	Sparague Dawley rat	Increase body weight, serum lipid, liver, and kidney damage	[99]
Sucrose	Sparague Dawley rat	Increase urea and creatinine level	[15]
HFCS	Sparague Dawley rat	Kidney failure	[103]
Sugar syrup	Sparague Dawley rat	Hypercholesterolemia, hypertriglyceridemia, and hyperinsulinemia	[104]
Sugar cane vinasse	Oreochromis niloticus (tilapia fish)	Liver damage	[34]
Fructose	Rat	Renal failure	[106]
Sugar cane extract	Broiler chicken	Hypertrophied intestinal villi and epithelial cells	[12]
High fructose	Sparague Dawley rat	Hypertriglyceridemia	[109]
